# Advances in Imaging of Inflammation, Fibrosis, and Cancer in the Gastrointestinal Tract

**DOI:** 10.3390/ijms232416109

**Published:** 2022-12-17

**Authors:** Kylene M. Harold, William M. MacCuaig, Jennifer Holter-Charkabarty, Kirsten Williams, Kaitlyn Hill, Alex X. Arreola, Malika Sekhri, Steven Carter, Jorge Gomez-Gutierrez, George Salem, Girish Mishra, Lacey R. McNally

**Affiliations:** 1 Department of Surgery, University of Oklahoma Health Sciences Center, Oklahoma City, OK 73104, USA; 2 Department of Bioengineering, University of Oklahoma, Norman, OK 73019, USA; 3 School of Medicine, Emory University, Atlanta, GA 30322, USA; 4 Department of Child Health, School of Medicine, University of Missouri, Columbia, MO 65211, USA; 5 Wake Forest Baptist Health, Winston-Salem, NC 27157, USA

**Keywords:** imaging, gastrointestinal tract, inflammation, fibrosis, cancer

## Abstract

Gastrointestinal disease is prevalent and broad, manifesting itself in a variety of ways, including inflammation, fibrosis, infection, and cancer. However, historically, diagnostic technologies have exhibited limitations, especially with regard to diagnostic uncertainty. Despite development of newly emerging technologies such as optoacoustic imaging, many recent advancements have focused on improving upon pre-existing modalities such as ultrasound, computed tomography, magnetic resonance imaging, and endoscopy. These advancements include utilization of machine learning models, biomarkers, new technological applications such as diffusion weighted imaging, and new techniques such as transrectal ultrasound. This review discusses assessment of disease processes using imaging strategies for the detection and monitoring of inflammation, fibrosis, and cancer in the context of gastrointestinal disease. Specifically, we include ulcerative colitis, Crohn’s disease, diverticulitis, celiac disease, graft vs. host disease, intestinal fibrosis, colorectal stricture, gastric cancer, and colorectal cancer. We address some of the most recent and promising advancements for improvement of gastrointestinal imaging, including unique discussions of such advancements with regard to imaging of fibrosis and differentiation between similar disease processes.

## 1. Introduction

Gastrointestinal disease includes a host of pathologies, often associated with inflammation, infection, fibrosis, hemorrhage, malignancy, or some combination thereof. Gastrointestinal disease is prevalent, resulting in an estimated annual 3.8 million hospitalizations and 255,407 deaths [1], with hemorrhage being the leading cause of associated hospitalization and colorectal cancer being the leading cause of associated death [2]. Shared signs and symptomology present challenges with regard to diagnosis and definitive treatment of gastrointestinal diseases. Priorities in the development of new diagnostic modalities and techniques should therefore include improved differentiation between similar disease processes, early diagnosis, and minimizing invasiveness of diagnostic technologies, ultimately leading to improved outcomes and minimal complications. Gastrointestinal disease diagnostics rely heavily on imaging modalities to provide insight into the macroscopic structural abnormalities associated with these pathologies. Colonoscopy has supplanted barium enema as a mainstay for assessment and diagnosis of lower gastrointestinal diseases. However, it nonetheless presents several challenges including risk of complications from anesthesia and bowel perforation, especially in pediatric patients and those with co-morbidities [3], as well as issues with patient compliance [4]. This has driven increases in use of technologies such as computed tomography (CT) and magnetic resonance imaging (MRI) which have shown recent potential through greater optimization of equipment settings, increasing their effectiveness and applicability. Further advancements in molecular imaging of gastrointestinal disease provide greater diagnostic information on a cellular and molecular level to aid in clinical decision-making. Utilizing advanced imaging modalities and applications is likely to reduce subjectivity in diagnostics, reduce the risk of observing a small area not representative of the affected region of the gastrointestinal tract, and maximize safety. In this review, we address many of the recent advancements regarding imaging of the gastrointestinal tract, including novel technologies or applications which allow for fibrosis identification and differentiation between similar disease processes.

## 2. Inflammation

Inflammation is caused by autoimmunity or an immune response to infection, injury, irritants, and other triggers. Inflammation and infection account for approximately one quarter of cancer-causing factors, impacting pathophysiology and progression through their participation in cell growth, survival, and metastasis [5,6]. A significant challenge faced by the field of inflammatory gastrointestinal disease diagnostics is differentiation between similar disease processes, such as ulcerative colitis and Crohn’s disease. Machine learning technologies, biomarkers, and other recent advancements discussed here aim to address this. While great strides have been made in gastrointestinal inflammation diagnostics, there is tremendous room for improvement to maximize safety, specificity, and sensitivity while minimizing subjectivity in the diagnostic process.

### 2.1. Inflammatory Bowel Disease

Inflammatory bowel disease refers to chronic gastrointestinal inflammation, encompassing ulcerative colitis and Crohn’s disease. While IBD can occur at any age, the peak age of onset occurs from 15–30 years of age [7]. It is estimated that up to 20% of people with IBD are diagnosed during childhood [8], and the incidence and prevalence of pediatric IBD is increasing worldwide [9]. Ulcerative colitis (UC) is a chronic and often intermittent or relapsing inflammatory disease, characterized by a host of non-specific symptoms. These include nausea, fatigue, weight loss, bowel obstruction, diarrhea, and abdominal pain [10]. Endoscopic signs include bleeding, ulcerations, granularity, and abnormal vascularity, among others [11]. The etiology of UC is suspected to be a combination of heritable, environmental, and immune factors with intestinal microbiota [12] and disruption by bacterial infection [13] also potentially playing a role. Additionally, cytomegalovirus increases severity of inflammation and other symptoms in the context of UC [14,15]. Currently, UC is primarily diagnosed by clinical signs and symptoms, exclusion of infection, and endoscopy with more definitive confirmation by biopsy and histopathology [16,17].

Crohn’s disease (CD) generally presents similarly to UC, with the primary distinction being the potential presence of lesions anywhere along the alimentary canal in CD [18] as opposed to the relative localization in the colon in UC. Although, disease is localized exclusively to the colon in 25% of CD patients [19]. The transmural nature of CD is also unique, predisposing CD patients to penetrating lesions or stenosis of the bowel, and indicating benefit of cross-sectional imaging modalities such as computed tomography and magnetic resonance imaging which can better assess this transmural nature [20]. In addition, cross-sectional imaging allows for assessment of small intestine not amenable to endoscopic evaluation.

Differences in treatment standards require differentiation between UC and CD, though this is not achieved in approximately 5% of patients with chronic inflammatory bowel disease who ultimately remain classified as “indeterminate colitis” [21]. A recently developed machine learning model may allow for more definitive diagnosis through utilization of RNA sequencing to differentiate between the two disease processes [22]. The disadvantage to this technique is the biopsy-associated risk, though biopsy is generally already a component of standard of care diagnostics. Nonetheless, there is certainly potential for development of a less invasive means of diagnosis and differentiation of CD and UC which is not reliant on biopsy. Aside from the biopsy-related risks associated with anesthesia or potential for bowel perforation, there is a tremendously increased risk associated with obtaining a biopsy on patients who potentially have bowel infections or are passing large volumes of diarrhea. Novel molecular biomarkers may provide a strong alternative, relying on samples such as blood and fecal matter which are non-invasive [23], and imaging technologies may hold promise in this area as well.

### 2.2. Diverticulitis

Diverticulitis, diverticular inflammation, is the leading cause of colon operations and gastrointestinal-related hospitalization [24,25]. It can be classified as uncomplicated or complicated, with complicated diverticulitis being characterized by abscess, phlegmon, perforation, obstruction, or bleeding [26]. In addition, there exists a potential for fistula or stricture formation. Due to the microbial nature of diverticulitis, antibiotics are often utilized in medical management of acute complicated diverticulitis, though surgical management is also common.

Diverticulitis patients often present with persistent left lower quadrant abdominal pain or tenderness, abdominal distention, and a host of non-specific abdominal symptoms. Diagnosis of the disease is generally through clinical signs and basic laboratory testing, including a complete blood count, C-reactive protein measurement, metabolic panel, and urinalysis, with computed tomography being used to further determine disease severity [27]. Diverticulitis is frequently misdiagnosed, demanding improvement in diagnostic capabilities. A combination of biomarkers, i.e., fecal calprotectin [28] and elevated C-reactive protein levels [29], with symptomatic presentation may reduce misdiagnosis [30]. To better overcome misdiagnosis of diverticulitis and IBD, use of newly emerging imaging technologies may also be helpful.

### 2.3. Celiac Disease

Celiac disease is a prevalent disease which is caused by genetic determinants and triggered by gluten consumption, impacting 0.5–1% of the population globally [31]. While multifaceted, celiac disease most often presents as an immune-mediated enteropathy triggered by development of gluten peptides subsequent to gluten consumption [32]. These immune responses ultimately result in small intestinal mucosal inflammation and damage as well as malabsorption in genetically predisposed individuals [33].

While celiac disease has effective diagnostic biomarkers, tremendous gaps in effective treatment remain. Diagnosis of celiac disease is generally achieved through serological results prior to and following a change to a gluten-free diet and through duodenal biopsy [31]. However, serology may be sufficient due to its prediction accuracy and minimal invasiveness, potentially eliminating the need for biopsy [34]. Novel biomarkers identify potential informants of celiac disease in response to gluten exposure, such as inflammatory plasma cytokines, with IL-2 being the most prominent and longstanding [35,36]. Overall, diagnosis of celiac disease largely depends upon biomarker assessment, eliminating the need for imaging modality use.

### 2.4. Imaging Inflammation

**Figure 1:**
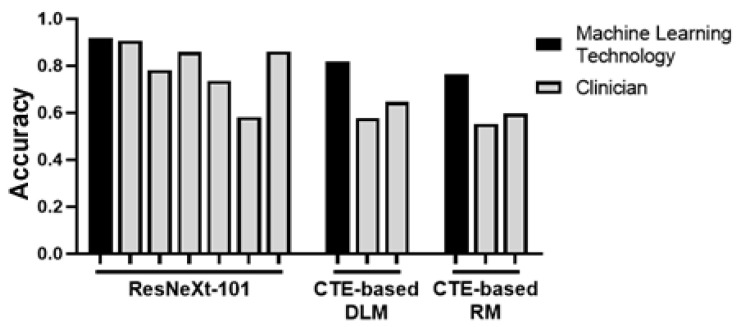
Machine learning- and clinician-derived data [38,62,63] were compiled into the depicted graph to demonstrate accuracy as measured by either percent or AUC. In each case, machine learning algorithms performed on par or better. This is anticipated to reduce human error in accurate diagnosis (original graph).

Colonoscopy is perhaps the most common and important mechanism by which inflammatory bowel disease is imaged and pathologically diagnosed [37]. However, extraordinary expertise and precision are required for proper interpretation of and differentiation between images from UC and CD patients. Recently, a convolutional neural network was established to mitigate this issue. ResNeXt-101 classified endoscopic images of CD, UC, and healthy bowel at an accuracy rate of 90.91% in per-patient analysis, superior to rates of a majority of clinicians, indicating its potential for future clinical application [38] (Table 1; Figure 1).

Similarly, a deep learning convolutional neural network-based diagnosis system has been developed based on endoscopic images and videos to score and predict gastrointestinal inflammatory activity [40]. However, applications of these deep learning advancements reach beyond endoscopy. Another recently developed system utilized multiple deep learning networks to classify intestinal inflammation based on micro-ultrasonography, allowing detection prior to human detectability by micro-ultrasound or endoscopy [75]. Machine learning opportunities to minimize inconsistency and remove subjectivity from the diagnosis process are becoming increasingly abundant (Figure 1).

The associated hallmarks of inflammation provide several surrogates for measuring inflammatory disease processes. In addition to endoscopy, CT enterography (CTE) is often used as a means of imaging IBD to identify increased thickening of the intestinal wall [11,41]. Similar parameters can also be measured using intestinal ultrasonography [76] and magnetic resonance enterography [77]. A recent development in the use of the latter involves utilization of very small superparamagnetic iron oxide nanoparticles (VSOPs) as a contrast agent to detect intestinal inflammation and extracellular matrix composition changes. The mechanism depends on the altered abundance of glycosaminoglycans such as hyaluronic acid in response to changes to the extracellular matrix and inflammation. These factors impact VSOP binding and, thus, MRI image enhancement by VSOPs [78]. ^18^F-fluorodeoxyglucose (FDG)-PET/CT and ultrasonography techniques can also be used to determine molecular information about the gastrointestinal tract including protein dysregulation [79], immune cell presence [80], or biochemical activity [81] through use of targeted contrast, all suggesting future improvements in these fields.

**Figure 2:**
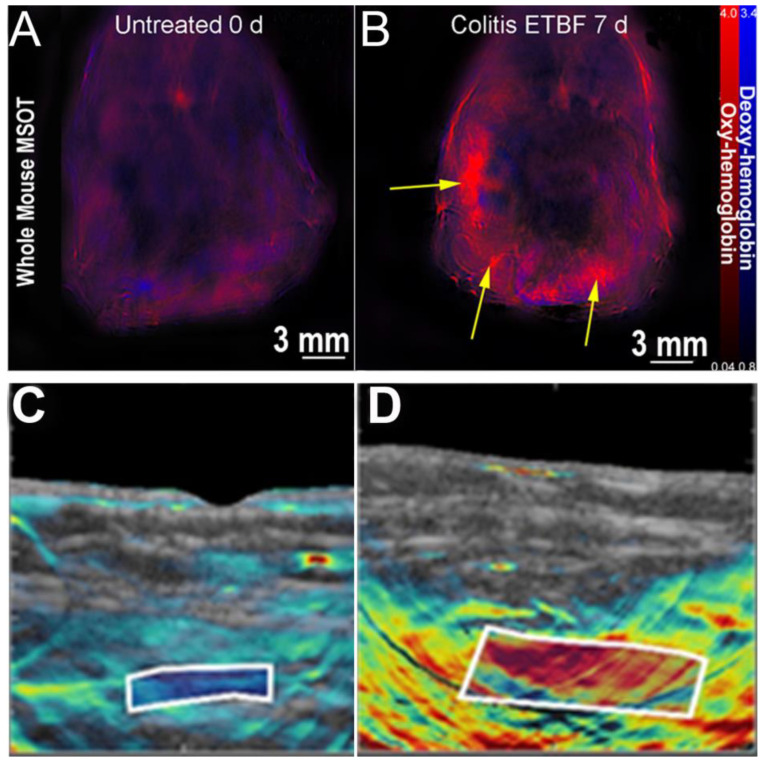
MSOT can visualize colitis (**A**,**B**) and Crohn’s disease (**C**,**D**) by utilizing differential levels of oxy- and deoxyhemoglobin. (**A**) Control murine model with no detectable areas of colitis. (**B**) Murine model treated with enterotoxigenic Bacteroides fragilis, resulting in visually concentrated areas of colitis (yellow arrows). (**C**) Intestinal wall of a patient in remission from Crohn’s disease, showing previous inflammation seen as deoxyhemoglobin in the white box. (**D**) Intestinal wall of a patient with active Crohn’s disease, showing inflammation seen as oxy-hemoglobin in the white box. Panels A and B are adapted with permission from [47]. Panels C and D adapted with permission from [86].

Multispectral optoacoustic tomography (MSOT) is a non-invasive imaging technology which utilizes near infrared light to reduce photon scatter compared to optical imaging modalities [82]. It thus provides increased imaging depth without the sacrifice of resolution observed in optical imaging modalities. Exogenous agents are utilized to visualize select chromophores in the NIR-II window for high-resolution imaging of ulcerative colitis [83] or microparticles within the gastrointestinal tract [84]. In addition to use of exogenous contrast agents, MSOT can provide visualization and quantification of endogenous contrast agents such as oxygenated and deoxygenated hemoglobin which serve as proxies for perfusion and, thus, inflammation [85]. MSOT has been shown to be effective in preliminary clinical and pre-clinical models of CD [48,86] and colitis [47,87] (Figure 2). While no studies to date have directly investigated the efficacy of MSOT in diverticulitis assessment, the properties and advantages of the optoacoustic effect suggest potential benefit for diagnostic application in determining diverticular inflammation.

## 3. Fibrosis

Fibrosis is the accumulation of extracellular matrix components, primarily collagen, in tissues of various organ systems, often leading to organ dysfunction and increased mortality. Fibrosis generally occurs in response to inflammation, cancer, and trauma, as well as heritable diseases such as cystic fibrosis. Gastrointestinal fibrosis and stricture often play a role in diseases such as Crohn’s disease [88,89]. Meanwhile, other gastrointestinal pathologies such as graft versus host disease (GvHD) or post-endoscopic submucosal dissection stricture are almost exclusively characterized by fibrosis. Historically, gastrointestinal fibrosis has been difficult to identify. However, recent imaging advancements provide promise to improvement of diagnostics and thus patient outcomes in this area.

### 3.1. Graft Versus Host Disease

**Figure 3:**
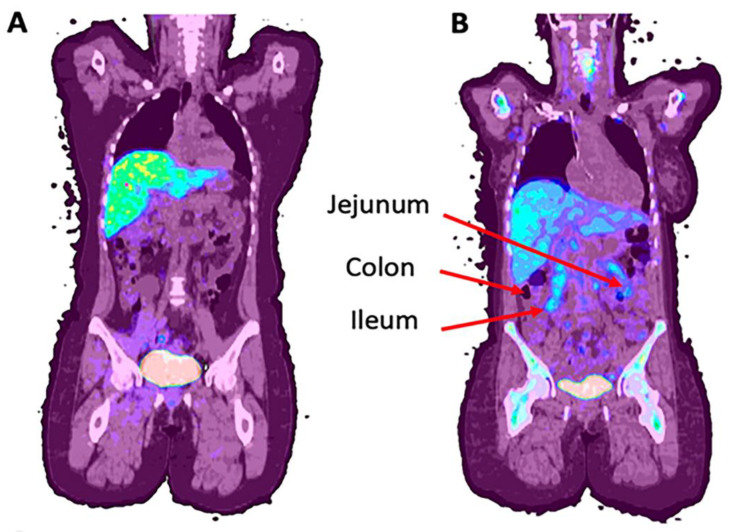
Examples of ^18^F-FLT uptake in patients with (**A**) no GvHD and (**B**) jejunum and ileum uptake c/w GvHD (NCT01338987). (Original data).

Graft versus host disease is a life threatening disease process in allogeneic hematopoietic stem cell transplantation as a result of donor T lymphocyte rejection of recipient tissue [90]. The risk of this increases in cases of greater HLA disparity between the donor and recipient, with other risk factors also having an impact [91]. GvHD is notably different from graft failure, the rejection of donor tissue by the host. Briefly, the pathophysiology of GvHD includes pre-transplant host tissue damage, followed by activation of donor T-cells and inflammatory factor release which results in amplified tissue damage [92,93]. This ultimately results in widespread inflammation and multiorgan system fibrosis [94]. One of the greatest challenges of GvHD is its nearly identical symptomatic presentation and appearance on most imaging devices to colitis. Further, the immune suppressants required for bone marrow transplantation put patients at greater risk of bacterial or viral-mediated colitis, such as cytomegalovirus-driven colitis. As both diagnoses are therefore likely and have divergent treatments, this diagnostic ambiguity can be associated with significant morbidity and mortality. A relatively new and currently accepted diagnostic approach, The Mount Sinai Acute GvHD International Consortium algorithm probability (MAP), utilizes serum biomarkers ST2 and REG3α to determine damage to intestinal crypts in the context of GvHD [95]. MAP is believed by many to be a superior prognostic indicator to previous clinical standards, Glucksberg criteria and the International Bone Marrow Transplant Registry severity index [96], which rely on patterns of organ involvement and, in the case of Glucksberg criteria, clinical performance [97,98]. However, while the MAP test may provide significant value in predicting non-relapse mortality, the acuity of the disease in many patients calls for a more rapidly and readily available means of obtaining diagnostics which can inform acute treatment. Recently, we have aimed to address this through use of ^18^F-fluorothymidine (^18^F-FLT) for GvHD assessment (Figure 3).

### 3.2. Intestinal Fibrosis and Colorectal Stricture

Intestinal fibrosis, specifically including colorectal strictures, can provide an extreme degree of patient discomfort, potentially detrimental to quality of life [99]. Due to the relationship between inflammation and fibrosis, colorectal strictures are not uncommonly observed in patients with inflammatory bowel disease or diverticulitis. Strictures can also occur in response to surgical intervention or ischemic events [100]. Yet another potential cause of intestinal stricture is procedural intervention. A prime example is that which occurs in response to endoscopic submucosal dissection (ESD), known as post-ESD stricture. ESD is a relatively recent advancement in gastrointestinal neoplastic therapy. Stenosis, among other complications, occurs much more frequently in ESD relative to endoscopic mucosal resection, the comparable technique [101]. However, these risks may be outweighed by the decreased recurrence rates following ESD procedures relative to endoscopic mucosal resection [102]. Nonetheless, the fibrosis resulting from the procedure can result in strictures. ESD procedures involving circumferential ESD or subtotal dissection of ≥90% of the rectal circumference serve as an independent risk factor for strictures [103]. This calls for an efficient and non-invasive means of monitoring stenosis of post-ESD lesions, as well as gastrointestinal tissue in patients with other risk factors, to allow for medical management when appropriate. Imaging may provide that monitoring mechanism.

### 3.3. Imaging Fibrosis

Though the gap in adequate technologies suitable for imaging fibrosis has historically been vast, a host of recent developments address this issue. While computed tomography enterography (CTE) is not a novel imaging modality for gastrointestinal disease diagnostics, various recent developments provide potential for improving its effectiveness as CTE was not previously a reliable technology for imaging fibrosis. In 2021, a radiomic model was developed which provided significantly greater accuracy in characterizing enteric fibrosis than human radiologist capability [62] (Figure 1). However, while development of a radiomic model made great strides in providing a method of evaluating intestinal fibrosis, it has limitations which were addressed by the investigators through another machine learning-based approach. As in intestinal inflammation, recent development of a deep learning system has also provided increased accuracy and objectivity in interpretation of intestinal fibrosis. Accuracy of fibrosis severity assessment by this novel CTE-based deep learning model was greater than CTE assessment by two radiologists [63] (Figure 1). Another recent study aimed to further characterize and resolve intestinal fibrosis grading, also utilizing CTE. The authors developed a nomogram which combined clinical markers and CTE-derived mesenteric abnormality findings, resulting in a successful differentiation model between severity levels of intestinal stenosis [104].

As with CTE, MRI has long been used for gastrointestinal imaging. Benefits of MRI include the ability to observe the bowel transmurally and from various perspectives. Recent advancements have made MRI increasingly effective for imaging fibrosis. For example, magnetization transfer imaging, a contrast mechanism sensitive to intestinal collagen, and native T1 mapping, a quantitative technique capable of identifying fibrosis characteristics, have both been established as promising advancements in the field of magnetic resonance with regard to bowel fibrosis detection and differentiation [64]. Another example involves use of diffusion weighted imaging. Diffusion weighted imaging capitalizes on the fact that when tissues are inflamed, diffusion of water molecules is restricted [43]. Mapping of water molecule diffusion is utilized, as quantified by the apparent diffusion coefficient (ADC). With regard to fibrosis, the apparent diffusion coefficient has been found to be significantly correlated with histopathologically derived inflammation and fibrosis scores, as well as percent gain. Further, based on an established cutoff value, the apparent diffusion coefficient correctly distinguished fibrosis with 72% sensitivity and 94% specificity, proving its potential usefulness as a non-invasive technology contributing to fibrosis identification [105]. Building upon this is diffusion kurtosis imaging which allows for identification of tissue diffusional heterogeneity through dimensionless quantification of deviation from Gaussian behavior [106]. A study which confirmed significant ADC correlation with histologically derived inflammation grades also showed that ADC and apparent diffusional kurtosis were significantly correlated to histologically derived fibrosis grades. The authors further showed that apparent diffusional kurtosis was able to differentiate absence of fibrosis or mild fibrosis from moderate to severe fibrosis with a sensitivity of 95.9% and specificity of 78.1%, indicating its potential for beneficial application to MRI use in bowel fibrosis assessment [61]. Additionally, the aforementioned MSOT imaging technology has potential for future intestinal fibrosis diagnostics due to the capability to detect collagen as a result of exhibited optoacoustic signal [107]. Overall, relatively new technologies, as well as numerous improvements and developments building on pre-existing technologies, have potential to revolutionize the field of imaging fibrosis, improving diagnostic outcomes for countless patients.

## 4. Cancer

Cancer is a disease marked by the toxic over-proliferation of cells within the body. Gastric cancer and colorectal cancer are both devastating diseases with complex, multifactorial etiologies including causal influences such as diet, bacterial infection, and pre-existing medical history. The biggest prognostic indicator for cancer is the stage at diagnosis, making it imperative that the disease is identified early and that the stage of disease is precisely determined. This staging uses a combination of biomarker testing and imaging techniques.

### 4.1. Gastric and Colorectal Cancer

Gastric cancer ranks fifth among cancers in diagnostic prevalence and third in mortality worldwide [108]. Anatomically, gastric cancer is observed as cardia or non-cardia by location in the uppermost and more distal portions of the stomach, respectively [109]. While there are some similarities between the etiologies of cardia and non-cardia gastric cancer, there are differences as cardia risk factors include obesity and gastroesophageal reflux disease [110], while non-cardia risk factors include dietary factors, atrophic gastritis, and *Helicobacter pylori* infection [110,111]. Gastric cancer patients commonly present with upper abdominal pain and weight loss, as well as potential nausea, melaena, or dysphagia [112]. Biomarker testing and pathology reports are often used as diagnostic tools along with traditional imaging technologies. Despite use of multiple standard imaging techniques, in many instances poor molecular understanding and inter-reader variability result in ultimate reliance on biopsy for improved diagnostic accuracy. Current diagnostic limitations result in approximately 50% of patients presenting with advanced stages of the disease at time of diagnosis and therefore having poor prognoses [108]. As technologies and contrast agents are developed further, improvements in diagnosis and monitoring of gastric cancer should follow.

Colorectal cancer (CRC) is the third most prevalent with the second highest mortality rate of cancers worldwide [113]. While the disease is decreasing in overall incidence due to improved screening, CRC is rising in the younger patient population [114]. CRC patients often present with abdominal pain, lack of appetite, constipation, diarrhea, abdominal distention, intestinal bleeding, and a host of non-specific symptoms [115]. There are many additional non-invasive biomarker screenings which can be performed using stool samples, including the fecal immunochemical test (FIT), high-sensitivity guaiac fecal occult blood testing (gFOBT), and multitarget stool DNA (mtsDNA) tests [116]. Using biomarkers in conjunction with one another, i.e., a combination of FIT and *Fusobacterium nucleatum* tests, provides improved accuracy of diagnosis with regard to sensitivity and specificity [117].

More specifically, rectal cancer is defined as adenocarcinoma cases arising within 15 cm from the anal verge. Rectal cancer comprises around 30–40% of colorectal cancer cases [118]. Successful management following endoscopic and radiographic staging is achieved through a multidisciplinary approach including appropriate surgical intervention. The prognosis is directly related to tumor extent, mesorectal infiltration, and achievement of disease-free circumferential surgical margins [119].

### 4.2. Imaging Gastrointestinal Tract Cancers

Colonoscopy is a standard of care technology in the area of colorectal cancer (CRC) screening, primarily due to its high sensitivity and specificity for detecting cancerous and pre-cancerous lesions [120]. Colonoscopy utilizes a flexible endoscope to evaluate the entire colon and rectum. Both colorectal cancer and adenomas ≥10 mm can be detected by colonoscopy with a sensitivity of approximately 95% [121]. Sigmoidoscopy, a technology which generally utilizes a similar preparation and mechanism to colonoscopy, can be performed without anesthesia or oral bowel preparation when necessary [122], circumventing some of the limitations of colonoscopy. However, as this modality is limited by failure to evaluate the proximal colon, patients with positive sigmoidoscopy results generally will require a colonoscopy follow up [123].

In gastric cancer, endoscopic ultrasound is a preferred modality for differentiation between submucosal and mucosal lesions due to its enhanced ability to distinguish distinct layers of the gastric mucosa relative to other modalities [124]. Notably, EUS has been demonstrated to distinguish T1 and T2 from T3 and T4 gastric cancers with 86% sensitivity and 90% specificity [125] and detect N stage with an accuracy of 76.2% for staging and 88.5% for restaging, significantly higher than that of PET-CT [67]. However, despite its strong ability to distinguish between the different gastric layers, EUS does have limitations, specifically with regard to invasiveness and potential for human error.

In addition to conventional endoscopic imaging methods, MSOT has the potential to expand into an endoscopic technique through development of specialized probes as evidenced by related applications in gynecological disease [126]. MSOT not only benefits from the aforementioned advantages related to circumventing photon scatter, but this technique may offer advanced functional information compared to EUS. As stated, MSOT has shown potential for imaging diseases that include fibrosis and inflammation due to differential oxy/deoxyhemoglobin and collagen concentrations, the most viable endogenous optoacoustic contrast agents [82]. This includes GI defects such as Crohn’s disease [48] and ulcerative colitis [87]. As evidenced by MSOT imaging in dermatologic cancer [127], breast cancer [128,129,130], and thyroid cancer [131], development of specific exogenous contrast agents will facilitate application of MSOT in a clinical setting for gastrointestinal cancer imaging.

Computed tomography remains a first line imaging modality in cancer patients and others presenting with abdominal pain. This is due in large part to its availability and ability to identify a wide variety of pathological changes [28], despite risks associated with ionizing radiation [132] and requirement for adequate gastric distention [133]. Recent improvements to the spatial resolution of CT, specifically with multidetector computed tomography (MDCT), prove to be beneficial in improving diagnostic accuracy, notably evident in locoregional staging. MDCT demonstrated a 76.9% diagnostic accuracy for T staging of gastric cancer compared to 74.7% by EUS in a comparative study, though EUS outperformed MDCT in N staging accuracy [134].

The specificity of CT in detection of regional and distal lymph node metastasis can be enhanced upon integration with ^18^F-fluorodeoxyglucose positron emission tomography (^18^F-FDG- PET). As CT detects lymph node metastasis based on anatomical abnormality, the presence of enlarged inflammatory nodes and minimally enlarged tumor-harboring lymph nodes can impair accuracy of detection [133]. Therefore, ^18^F-FDG PET/CT is able to further inform the diagnostic process as compared to routine CT alone. Though less sensitive than conventional CT, the increased specificity provided by ^18^F-FDG PET/CT has potential to reduce unnecessary interventions [69].

**Figure 4:**
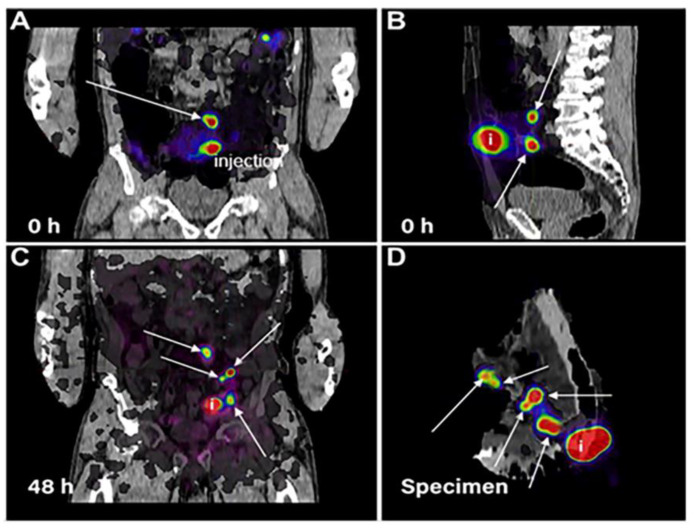
PET-CT identification of sentinel lymph nodes following injection of colloidal albumin radiolabeled with ^89^Zr. Arrows indicate positive disease. Top images of the frontal (**A**) and sagittal (**B**) plane immediately following injection of radiotracer show foci. By 48 h post-injection (**C**), lymph node foci are clearly visible, prior to surgery. Static PET-CT of the specimen following surgery corroborates pre-operative images (**D**). i represents location of injection. Reprinted/adapted with permission from [135].

In addition to ^18^F-FDG PET, other PET techniques have been explored for gastric imaging. A recent alternative method for assessment of tumor-positive sentinel lymph nodes includes a colloidal solution of albumin which has been labeled with ^89^Zr for preferential uptake in sentinel lymph nodes, detectable with PET/CT. The study yielded clear visualization of cancerous foci, providing the surgeon with valuable information pre-operatively to ensure complete removal of cancerous lesions (Figure 4) [135].

**Figure 5:**
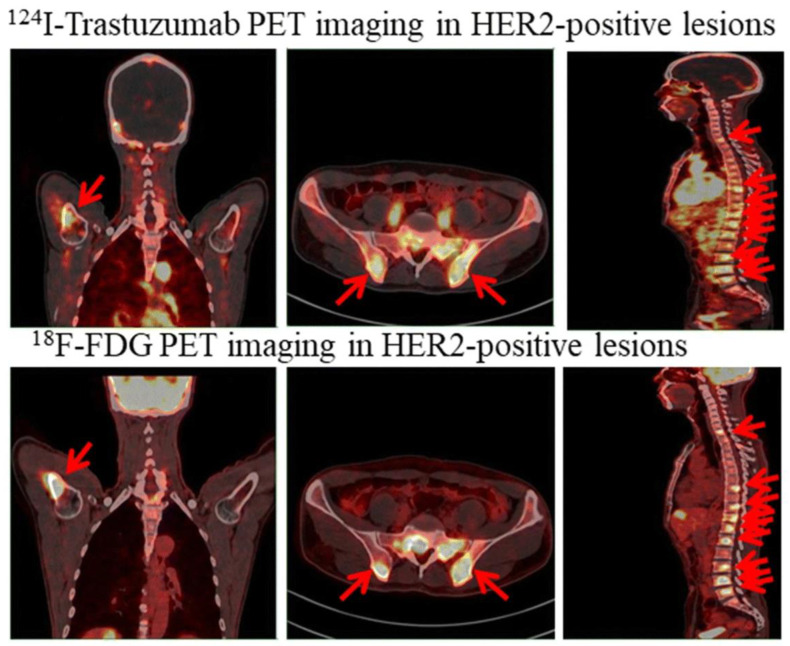
Detection of HER2-positive lesions with ^124^I-trastuzumab and standard ^18^F-FDG PET imaging in humeral, bilateral ilium, and spinal metastases. Arrows point to HER2-positive gastric cancer lesions. Reprinted/adapted with permission from Ref. [136].

An additional example of PET imaging that differs from conventional ^18^F-FDG-based contrast is the use of radiolabeled I^124^-trastuzumab. I^124^-trastuzumab undergoes preferential uptake in HER2-positive tumors relative to HER2-negative ones, allowing for visualization of HER2 positivity in both primary and metastatic lesions in gastric cancer patients using PET imaging [136] (Figure 5). Yet another PET application, the aforementioned ^18^F-FLT imaging, has been utilized in imaging of proliferative processes, specifically in cancer and to identify repopulation in proliferative systems such as the hematopoietic and lymphocyte systems [137,138,139,140]. Touted as a major advantage over ^18^F-FDG, ^18^F-FLT does not identify predictably inflammatory events in differentiated hematopoietic cells such as neutrophils [141]. However, ^18^F-FLT does identify certain disease processes that have been considered inflammatory systems. Furthermore, in the explosion of therapies that modulate the immune system, including drugs such as checkpoint inhibitors, ^18^F-FLT imaging has been useful to identify strong lymphocyte proliferation associated with immunologic response [138,142,143]. Utilization of ^18^F-FLT as a modality to evaluate and monitor response of lymphocytic systems continues to expand and ^18^F-FLT could be utilized as an imaging biomarker of lymphocytic inflammatory response via identification of lymphocyte proliferation [144]. Overall, while PET imaging is not without its disadvantages, namely high expense and low sensitivity, it provides unique benefits to the diagnostic process [132,145].

With regard to rectal cancer specifically, the initial local staging after endoscopic evaluation is performed using either magnetic resonance imaging (MRI) with rectal protocol, using thin-section MRI with pelvic phased-array coil, or transrectal ultrasound (TRUS) which is also known as endorectal ultrasound [119,146]. While, overall, TRUS represents a strong staging option, it is limited by the patient’s active symptoms, the tumor characteristics and bulkiness, tumor location [147,148], and the inherent operator-dependent nature of the procedure [149]. Other modalities such as MDCT may also be used to image rectal cancer. However, although MDCT is often used in evaluation of metastatic rectal lesions, its applicability for local rectal cancer staging has been challenged due to its limited sensitivity in resolving bowel wall layers compared to other modalities. Therefore, it is considered to be “usually not appropriate” as a modality for locoregional rectal cancer staging based on the American College of Radiology (ACR) appropriateness criteria for pre-treatment staging [150].

Radiomics is an increasingly attractive artificial intelligence-based imaging application for rectal cancer in particular, and can be applied to multiple different imaging modalities [151]. Use of radiomic features on MRI images improves sensitivity and specificity to 100% and 91%, respectively, while demonstrating a positive predictive value of 72–92% and negative predictive value of 96–100% when used to assess treatment response [152,153]. Radiomics offers a non-invasive option and the ability to obtain high-quality imaging, improving upon conventional MRI use in terms of lesion characterization, detection of pre-treatment rectal cancer pathological feature biomarkers [154], and post-treatment surveillance [152].

## 5. Conclusions

Gastrointestinal disease, specifically with regard to its inflammatory, cancerous, and fibrotic manifestations, is a significant issue with regard to hospitalizations, patient wellbeing, financial implications, and more. The increasing prevalence of inflammatory gastrointestinal diseases such as IBD is particularly striking, as observed in both adult and pediatric patients. An ongoing clinical challenge involves refining the diagnostics of these disease processes, related to both detection and, in many instances, differentiation. This is further complicated by the co-presentation of many of these disease processes, making both development and use of these differential diagnostic mechanisms difficult. Use of biomarkers and up-and-coming applications of imaging technologies prove to be promising in mitigating this issue. In the future, the imaging technologies discussed in this review, among others, could be combined in a multimodal context to provide a better picture of a given pathology. Further, multiplexed diagnostics with these imaging technologies and biomarkers or other diagnostic methods could provide an even greater comprehensive analysis of patients’ individual disease processes.

## Figures and Tables

**Table 1:** Summary of pre-existing and newly developed imaging modalities for inflammation, fibrosis, and cancer in the GI tract.

Disease		Modality	Citation
Inflammation	IBD	Endoscopy and Convolution Neural Network (CNN)	[38]
		Endoscopy	[39]
		Endoscopy and Deep Learning CNN	[40]
		Computed Tomography (CT) Enterography	[41]
		Magnetic Resonance (MR) Enterography	[42]
		MR Enterography: Diffusion Weighted Imaging	[43]
		Chromoendoscopy	[44]
		Transabdominal Ultrasound	[45]
		^18^F-FDG Positron Emission Tomography (PET)/MR Enterography	[46]
		Multispectral Optoacoustic Tomography (MSOT)	[47] [48]
	Diverticulitis	Ultrasound	[49]
		Colonoscopy	[50]
		Magnetic Resonance Imaging (MRI)	[51]
		CT	[52]
	Celiac Disease	Endoscopy	[53]
		CT	[54]
		Ultrasound	[55]
Fibrosis	Graft versus Host Disease	^18^F-FDG PET	[56]
		MRI	[57]
		^18^F-FDG PET and MRI	[58]
		Ultrasound	[59]
		CT	[60]
	Intestinal Fibrosis	MRI: Diffusion Kurtosis Imaging	[61]
		CT Enterography: Radiomic Model	[62]
		CT Enterography: Deep Learning Model	[63]
		Magnetization Transfer Imaging and Native T_1_ Mapping	[64]
Cancer	Gastric Cancer	Multidetector Row Computed Tomography	[65]
		MRI	[66]
		Endoscopic Ultrasound	[67]
		^18^F-FDG-PET/CT and Laparoscopy	[68]
		^18^F-FDG-PET/CT	[69]
	Colorectal Cancer	Colonoscopy and Sigmoidoscopy	[70]
		^18^F-FDG-PET/CT	[71]
		CT	[72]
		CT + Artificial Intelligence (AI) and MRI + AI	[73]
		MRI	[74]

## Data Availability

Not applicable.
